# Conversion of a ventricular rickham reservoir to a subcutaneous chest port for repeated intraventricular enzyme replacement therapy: technical note and surgical considerations

**DOI:** 10.1007/s00381-026-07463-x

**Published:** 2026-09-18

**Authors:** Mahie Gopalka, Samiya Manocha, Kathleen Romanski, Erin Hoeman, Scott Boop, Sandi K. Lam

**Affiliations:** 1 Department of Neurosurgery, Northwestern University Feinberg School of Medicine, Chicago, IL USA; 2 Division of Pediatric Neurosurgery, Ann & Robert H. Lurie Children’s Hospital, Chicago, IL USA

**Keywords:** CLN2 disease, Cerliponase alfa, Intraventricular infusion, Ventricular access device, Chest port, Pediatric neurosurgery

## Abstract

Cerliponase alfa enzyme replacement therapy has transformed the management of neuronal ceroid lipofuscinosis type 2 (CLN2) disease but requires lifelong cerebrospinal fluid (CSF) access for repeated intraventricular infusions. Conventional scalp-based ventricular reservoirs are associated with complications including infection, mechanical deterioration, and device revision related to repeated puncture. Chest port–mediated ventricular access has emerged as a potential alternative; however, detailed operative descriptions remain limited. We describe operative considerations and stepwise technique for establishing ventricular access using a Rickham reservoir connected via shunt tubing to a subcutaneous chest port system for repeated intraventricular cerliponase alfa infusion. Illustrative clinical experience demonstrates durable long-term use of this configuration. Key technical elements include neuronavigation-guided ventricular catheter placement, creation of a subcutaneous or subfascial chest pocket depending on patient body habitus, incorporation of strain-relief loops to reduce catheter tension, and systematic testing of the completed construct. The resulting system allows reliable ventricular access via a chest port while preserving ventricular catheter integrity. Chest port–mediated ventricular access represents a feasible and durable strategy for long-term intraventricular therapy in CLN2 disease. Dissemination of operative technique may facilitate broader adoption and support reliable delivery of enzyme replacement therapy for patients requiring lifelong treatment.

## Introduction

Neuronal ceroid lipofuscinosis type 2 (CLN2) disease is a progressive lysosomal storage disorder caused by deficiency of tripeptidyl peptidase-1, resulting in neurodegeneration, seizures, motor decline, and premature death in childhood [2, 11]. Intraventricular cerliponase alfa enzyme replacement therapy has altered the natural history of CLN2 by slowing neurologic deterioration and improving survival outcomes compared with untreated cohorts [6, 14, 15]. Effective therapy requires direct intracerebroventricular administration to achieve adequate central nervous system distribution, necessitating lifelong biweekly cerebrospinal fluid (CSF) infusions through implanted ventricular access devices [1, 8].

Reliable and durable ventricular access is therefore a critical component of CLN2 management. However, conventional scalp-based ventricular reservoirs are associated with device-related complications including infection, leakage, obstruction, and mechanical degradation related to repeated puncture, frequently requiring revision or replacement [4, 5, 10, 15].

Recent series including that from our team in Boop et al. have demonstrated that connecting ventricular catheters to subcutaneous chest port–mediated ports provides reliable long-term intraventricular access with improved procedural usability and favorable safety profiles with no infections, device failures, or revisions and hundreds of successful infusion accesses [3, 13]. Implantable chest ports are designed for repeated access with noncoring (Huber) needles and have been reported to withstand approximately 1000–2000 punctures depending on device specifications [9]. In contrast, conventional Rickham reservoirs are susceptible to structural deterioration following repeated needle access, including fluid leakage secondary to reservoir fracture; in clinical practice, durability is generally estimated at approximately 100 punctures, with published literature reporting revision after repeated access [4].

Similarly, comparative analyses have demonstrated improved usability of chest-based devices, including higher first-attempt access success, reduced sedation requirements, and improved provider satisfaction, while maintaining complication rates comparable to traditional head-based reservoirs [13]. Securement of the Huber needle for infusion at a chest port is also perceived to be advantageous for the patient family, given improved durability, visualization, and needle stability at the access site because the chest location avoids interference from scalp hair and the option for clear adhesive tape rather than head wrapping to assist with needle stability over long infusion times [7].

To our knowledge, detailed operative guidance describing technical considerations for chest port–mediated ventricular access has not previously been published. In this technical note, we describe operative considerations and stepwise technique for establishing ventricular access using a subcutaneous chest port system for repeated intraventricular cerliponase alfa infusion.

## Illustrative case

A pediatric patient diagnosed with CLN2 disease is indicated for chronic intraventricular cerliponase alfa therapy. We have implanted this chest port–mediated ventricular access configuration in a patient as young as 9 months old at the time of presymptomatic genetic diagnosis, with 5-year durability with regular infusion therapies. We know of no specific contraindications to this technique so far.

## Surgical technique

### Preoperative preparation

**Fig. 1:**
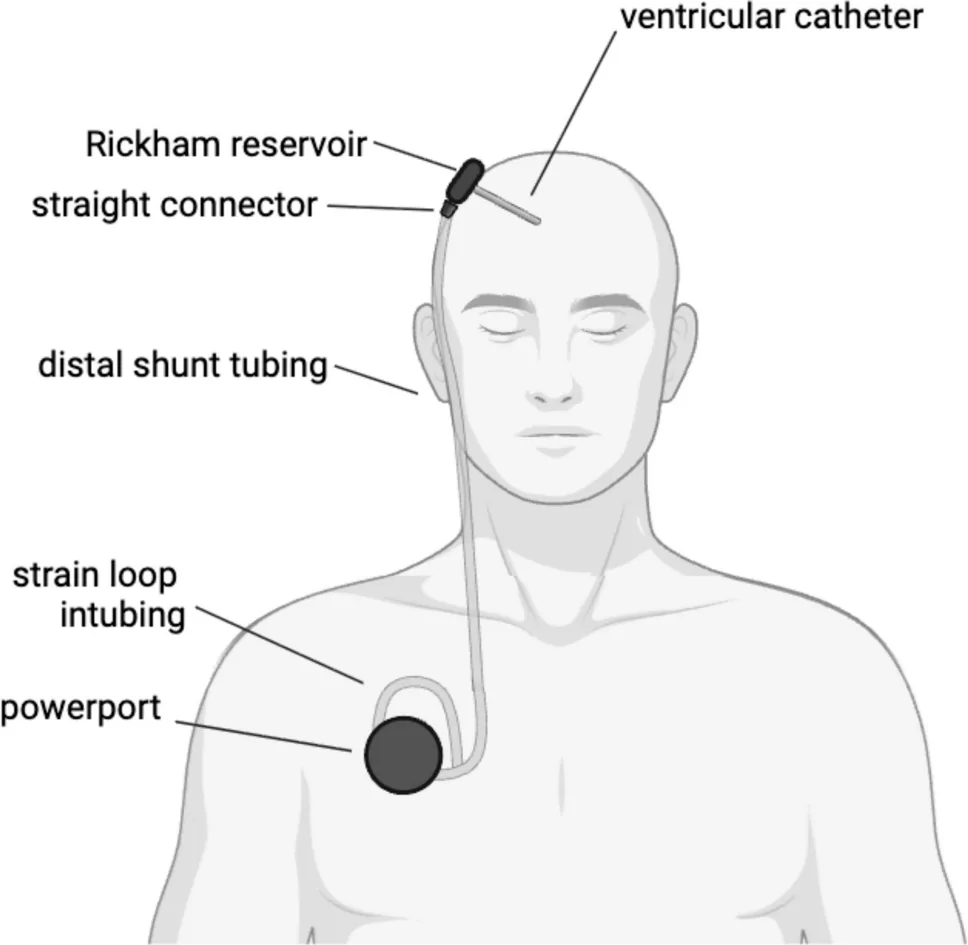
The construct consists of a Rickham ventricular reservoir connected via straight connector and distal shunt tubing to a subcutaneous chest port, with incorporation of a strain-relief loop

Under general anesthesia, electromagnetic neuronavigation is registered. The frontal entry site is selected anterior to the coronal suture in the midpupillary line with a trajectory planned in the navigation system toward a target at the foramen of Monroe. The chest wall incision is parallel to and two fingerbreadths inferior to the clavicle (Fig. 1).

### Chest port pocket creation

At the chest wall, a subcutaneous pocket is created, typically overlying the pectoralis muscle. For patients with limited subcutaneous fat, the depth can be modified to be subfascial. Hemostasis is essential. The pocket is sized to allow secure placement of an infusion port with all hardware positioned inferiorly well away from the incision.

### Cranial exposure and ventricular access

A frontal curvilinear incision is designed for a well-healing scalp flap to have no part of the incision overlying the hardware. Using neuronavigation guidance, the planned ventricular trajectory targets the foramen of Monroe of the lateral ventricle, with the entry site at the burr hole.

### Subcutaneous tunneling and system assembly

**Fig. 2:**
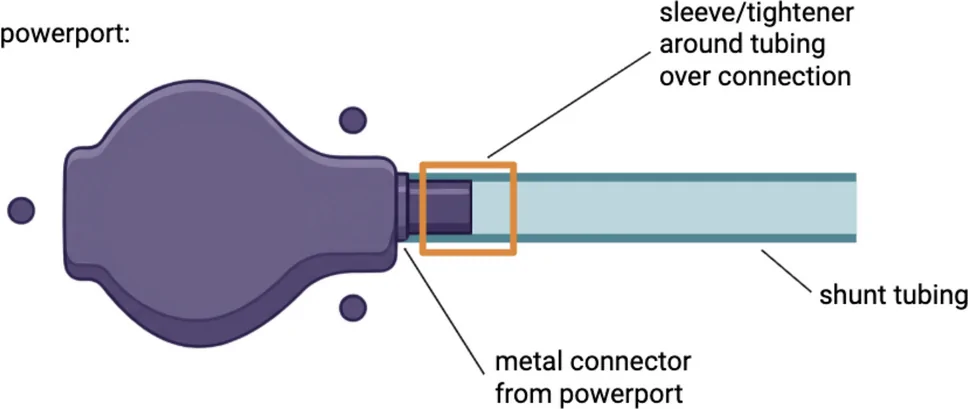
The connection between the chest port and distal tubing can be secured with the attached metal connector and protective tightening sleeve included in the chest port packaging

With a tunneler, distal shunt tubing is drawn through from the chest incision to a releasing incision up to the frontal cranial incision. The tubing is connected to the subcutaneous chest port using the attached metal connector with a protective tightening sleeve (Fig. 2). Depending on patient age at surgery, a strain-relief loop or even double loop (typically 5–10 cm) is incorporated in the tubing to allow for patient growth and movement. Typically, approximately 40–50 cm of total catheter length is implanted, with excess length coiled in the strain-relief loop. The chest wall incision should not be overlying the port.

### Ventricular catheter and reservoir management

Dural opening should be only as big as the catheter to prevent CSF leakage. The ventricular catheter is placed with neuronavigation to the target with confirmation of cerebrospinal fluid (CSF) return. While stereotactic neuronavigation is used for catheter placement in this technique, other adjunct strategies for surgical placement of the ventricular catheter exist—such as use of ultrasound visualization in children with open fontanelle or via the utilization of a ventricular access guide at established anatomical locations [12]. The catheter is connected to a Rickham ventricular reservoir with a patent outflow, taking care to prevent excessive CSF loss.

The tubing is primed with sterile saline. The proximal end of the tubing is connected to the patient outflow end of the Rickham reservoir with straight connector.

There is no official guidance on catheter type for this surgical configuration. To minimize any potential interactions between any impregnated catheter materials and the enzymatic therapy, we choose to use nonimpregnated catheters.

### System testing

The implanted system is tested by chest port–mediated access with a noncoring needle and verifying ability to withdraw CSF with a small syringe and to flush a small amount of sterile saline. The total system tubing length and/or CSF volume within the system is noted, which gives information of the volume contained in this construct for future infusions.

In regard to dosing of the medication, we calculate the specific length of the tubing used in the surgery for each patient, along with the known inner diameter of the tubing, to understand the volume necessary. Typically, this is less than 1 cc of fluid depending on specific catheter inner diameter and tubing length used and can be calculated via a simple cylinder volume calculation (volume = π * radius^2^ * height). For example, our most commonly used catheters have an inner diameter of 1 mm and typically 40–50 cm of catheter length is implanted, resulting in 0.31 to 0.39 cc of volume in the system. Our protocol uses 0.5–1 cc of the intraventricular electrolyte solution flush at the conclusion of every infusion. This is ample to flush through the entire system without alteration to the infusion protocol.”

### Postoperative management

Wounds are closed in standard fashion. The chest port is accessed for intraventricular infusions after appropriate healing 1–2 weeks after surgery.

### Radiologic follow-up

Akin to a ventriculoatrial shunt, it can be worthwhile to plan for follow-up X-ray imaging after 2 years of age if implanted prior to that age. With regard to the strain loop, since accrual of height is typically most at the long bones and less at cervical spine of the axial skeleton, the strain loop has been shown to be sufficient in our experience.

## Discussion

The operative technique described in this report provides detailed procedural considerations intended to facilitate safe and reproducible implementation of chest port–mediated ventricular access. Key technical elements include incorporation of strain-relief catheter loop to afford additional length of tubing and allow for patient movement and growth, positioning of incisions away from underlying hardware, and system assembly of catheter and port components. These procedural details are designed to reduce mechanical stress along the catheter tract and optimize infusion reliability.

This technical note fills a gap: it provides a practical framework of procedural steps, components, and nuances; it is by definition descriptive. Small series and their comparative outcome data are published elsewhere in the literature. Additional studies are needed to further evaluate long-term device survival, complication rates, and patient-centered outcomes associated with chest port–mediated ventricular access systems. Dissemination of this practical illustration expands this option of chest port–mediated ventricular access to the neurosurgery and patient community.

## Conclusion

The operative technique described in this report offers stepwise procedural guidance for implantation of a chest port–mediated ventricular access system for long-term repeated intraventricular infusion. Continued refinement of ventricular access methods may improve treatment reliability, reduce procedural burden, and enhance long-term care for children with CLN2 disease.

## Data Availability

No datasets were generated or analysed during the current study.
